# Biomimetic Composite Scaffold Based on Naturally Derived Biomaterials

**DOI:** 10.3390/polym12051161

**Published:** 2020-05-19

**Authors:** Ionela Andreea Neacsu, Adriana Petruta Serban, Adrian Ionut Nicoara, Roxana Trusca, Vladimir Lucian Ene, Florin Iordache

**Affiliations:** 1 Department of Science and Engineering of Oxide Materials and Nanomaterials, Faculty of Applied Chemistry and Materials Science, University Politehnica of Bucharest, 060042 Bucharest, Romania; neacsu.a.ionela@gmail.com (I.A.N.); adrian.nicoara@upb.ro (A.I.N.); vladimir.l.ene@gmail.com (V.L.E.); 2 National Research Center for Micro and Nanomaterials, Faculty of Applied Chemistry and Materials Science, University Politehnica of Bucharest, 060042 Bucharest, Romania; truscaroxana@yahoo.com; 3 Department of Chemical Thermodynamics, “Ilie Murgulescu” Institute of Physical Chemistry, Romanian Academy, 060021 Bucharest, Romania; 4 Department of Biochemistry, Faculty of Veterinary Medicine, University of Agronomic Science and Veterinary Medicine, 011464 Bucharest, Romania; floriniordache84@yahoo.com

**Keywords:** biocomposite, scaffold, eggshell membrane, chitosan, gelatin, hydroxyapatite, bone ash

## Abstract

This paper proposes the development of a biomimetic composite based on naturally derived biomaterials. This freeze-dried scaffold contains a microwave-synthesized form of biomimetic hydroxyapatite (HAp), using the interwoven hierarchical structure of eggshell membrane (ESM) as bio-template. The bone regeneration capacity of the scaffold is enhanced with the help of added tricalcium phosphate from bovine Bone ash (BA). With the addition of Gelatin (Gel) and Chitosan (CS) as organic matrix, the obtained composite is characterized by the ability to stimulate the cellular response and might accelerate the bone healing process. Structural characterization of the synthesized HAp (ESM) confirms the presence of both hydroxyapatite and monetite phases, in accordance with the spectroscopy results on the ESM before and after the microwave thermal treatment (the presence of phosphate group). Morphology studies on all individual components and final scaffold, highlight their morphology and porous structure, characteristics that influence the biocompatibility of the scaffold. Porosity, swelling rate and the in vitro cytotoxicity assays performed on amniotic fluid stem cells (AFSC), demonstrate the effective biocompatibility of the obtained materials. The experimental results presented in this paper highlight an original biocomposite scaffold obtained from naturally derived materials, in a nontoxic manner.

## 1. Introduction

In recent decades, the treatment of bone defects has become a severe problem because of the increasing incidence of different types of accidents and diseases. Moreover, the use of unfit treatment methods can produce even more complex issues. Allografts and autografts present major disadvantages, like lack of donors, donor morbidity site or immunogenic reactions [1,2]. The majority of accidents and diseases cause bone defects that cannot heal by themselves, so the development of more efficient treatments is still necessary. Through tissue engineering, one can develop tridimensional systems that could heal and favor the formation of new bone, termed scaffolds. A scaffold has to be biocompatible, biodegradable and its degradation byproducts must not be toxic to the human body. Additionally, in the case of bone regeneration, scaffolds must possess other important properties like osseointegration and osseoconduction [3,4,5]. In fact, natural bone itself is a composite made out of a main inorganic part (hydroxyapatite), an organic part (type I collagen, lipids, noncollagenic proteins), and water [6,7]. Bone regeneration scaffolds have been synthesized through different methods, using a wide variety of biomaterials, on both solid (porous) and injectable forms [8]. Since they are not permanent implants, their main role is to facilitate the formation of extracellular matrix that will substitute the scaffold in time. Each form has its own advantages; while injectable systems based on natural polymers are a minimally invasive solution in the case of surgical approaches, the highly porous tridimensional architecture of the scaffold assists the cellular attachment, proliferation and differentiation [9]. The developed system should be easy to manufacture in different shapes and sizes so it will allow an adequate in situ treatment of different bone defects for patients [10]. Additionally, the scaffold must promote the formation of new blood vessels that may transport nutrients and oxygen towards the defect site. In addition to these properties, the most important one is the potential to mimic the native bone. The natural bone architecture implies that hydroxyapatite crystals are found in between collagen molecules. A composite material that includes dispersed hydroxyapatite powder into natural polymers enhances the biomimetism of the system in terms of composition and structural resemblance to the extracellular matrix of bone [11,12].

Calcium phosphates (especially hydroxyapatite) have a high affinity for BMP (bone morphogenetic proteins), can osseointegrate, osseoconduct, and favor new bone tissue formation; therefore, they have been the main component of numerous studies, along with natural polymers (e.g., collagen, chitosan, gelatin and silk) [13,14]. The major disadvantages of these composite scaffolds are their slow biodegradability, reduced mechanical properties and potential toxicity, which restrict their clinical applications [15,16,17]. Chitosan (CS) possesses a similar structure to that of the glycosaminoglycans from the extracellular matrix, their main function being the facilitation of cellular adhesion. It is also biocompatible, biodegradable and presents good intrinsic antibacterial properties [17,18]. Gelatin (Gel) is a biocompatible, biodegradable polymer that is also used in bone tissue engineering. This biopolymer presents minimal antigenicity, is highly available, and possesses many Arg-Gly Asp sequences that promote the adherence of cells [18,19]. Moreover, it has cytokine leukemia inhibitory factor, which supports stem cell growth and proliferation [20].

Several studies regarding composites based on hydroxyapatite, chitosan and gelatin can be found in the literature. Most of them are focused on innovative scaffold fabrication techniques [21], use of commercial synthetic nano-hydroxyapatite [22], or tricalcium phosphate (TCP) as the inorganic compound [23]. As an alternative to commercial HAp, bone ash (BA) represents a natural source of both hydroxyapatite and TCP, with osseoconductive properties that favor its use as filling material for bone defects, healing and regenerating the hard tissue. The addition of bovine bone ash to a composite scaffold containing hydroxyapatite is expected to produce an even higher degree of biocompatibility, due to TCP presence, as several studies indicate [24,25]. In comparison to synthetic hydroxyapatite, the one obtained from natural sources is nonstoichiometric, because it can contain other elements besides Ca and P, like Na, Zn, Mg, K, Si, Ba or F. The presence of these elements in the hydroxyapatite composition makes it fairly similar to the one present in natural human bone [26,27].

Usually, natural materials present unique structures that cannot be replicated through conventional synthesis methods. Their pores, interconnected channels and interwoven structures make these materials appropriate candidates for natural synthesis templates [28]. The growth of hydroxyapatite particles on the micronic interwoven structure of the eggshell membrane (ESM) represents a relatively new and less studied approach towards proper restoration of hard tissue, recently presented by Sabu group and others [29,30,31]. Compared with other reported natural bio-templates for biomorphic synthesis of hydroxyapatite (bamboo, rattan [32]), ESM is widely available, considered waste, and is a source of natural collagen, chondroitin and hyaluronic acid [33]. Porcine small intestine submucosal (SIS) membrane was also recently reported as bio-template, due to its complex architecture of type I collagen fibers, but the obtained results do not demonstrate its ability to induce in vivo biomineralization [34].

Several studies present the synthesis of HAp powder based on ESM, but there are only a few regarding its further use in composite scaffolds. Among the latter, chitosan-nano hydroxyapatite scaffolds using ESM as bio-template were developed for dental tissue engineering [35], but no similar chitosan-gelatin composites have been reported, to the extent of our knowledge. A hydroxyapatite–gelatin–chitosan–fibrin–bone ash scaffold with excellent results was previously obtained, but the authors used a biological synthesis of HAp through microbial phosphatase enzymes, rather than using an ESM bio-template assisted method. The main inorganic compound in both cases was HAp, but it differs severely from a structural point of view, depending on the synthesis route [36].

Taking into account the potential characteristics mentioned above regarding HAp, gelatin, bone ash, and chitosan, the present study aims to develop biomimetic HAp by microwave synthesis in the presence of ESM as bio-template, then further incorporate it, along with bone ash, for the first time, into a mixt chitosan-gelatin polymeric matrix. The obtained scaffold is studied from both physicochemical and biological point of view, assessing its architecture and in vitro biocompatibility.

## 2. Materials and Methods

### 2.1. Materials

The commercial chemical synthesis reagents were hydrochloric acid (HCl, 37 wt.% in H_2_O, 99.999%), orthophosphoric acid (H_3_PO_4_, 85 wt.% in H_2_O, 99.99%), acetic acid (glacial, ≥99.7%), calcium nitrate tetrahydrate (Ca(NO_3_)_2_·4H_2_O, ≥99.0%), ammonium hydroxide solution (NH_3_ aq., 30 wt.%) chitosan (medium molecular weight, 75%–85% deacetylation degree), and gelatin, all purchased from Sigma-Aldrich (Darmstadt, Germany) and used without further purification. The various diluted solutions were made using ultrapure water. Tibia bone from a calf was received as a waste product from a local abattoir (Dragalina, Romania). Chicken eggshells were collected as waste from a local poultry farm (Crevedia, Romania).

### 2.2. Hydroxyapatite Synthesis

The hydroxyapatite synthesis by microwave heating was performed according to the method presented by Sabu et al., with a variation in the microwave exposure time of the final membranes [29]. The eggshells were immersed in 100 mL HCl 1M solution to facilitate the detachment of the eggshell membranes (ESM) from the shell. ESM were thoroughly washed with distilled water and left out to dry at room temperature for 24 h. 100 mL of H_3_PO_4_ 0.6M solution was prepared by the addition of 4 mL H_3_PO_4_ 85% in 96 mL ultrapure water. ESM were immersed in this solution for another 24 h in order to be impregnated with PO_4_^3−^ ions. The following step consisted of the absorption of Ca^2+^ ions onto the bio-template. Hence, 100 mL of Ca(NO_3_)_2_·4H_2_O 1M solution was prepared by the addition of Ca(NO_3_)_2_·4H_2_O in ultrapure water. The Ca/P ratio was maintained at 1.67, specific to hydroxyapatite. The calcium nitrate solution was added drop by drop under continuous magnetic stirring over the ESM and orthophosphoric acid mixture, then left to rest for an additional 24 h. The pH value of the obtained mixture was measured and stabilized at 10 with the addition of an appropriate amount of ammonium hydroxide solution. The membranes impregnated with the hydroxyapatite precursor ions were extracted and left out to dry at room temperature for 12 h. Finally, the membranes containing phosphate and calcium ions were thermally treated in a conventional microwave oven for 15 min at 700 W and 2.45 GHz. These calcined membranes were grinded, and biomorphic hydroxyapatite powder was formed—HAp (ESM).

### 2.3. Biomimetic Composite Scaffold Synthesis

The tibia bone was first cut into smaller pieces, boiled in water in order to remove all the exterior organic parts (residual meat), then thermally treated to form bone ash at 750 °C for 6 h. The resulting fragments were then automatically grinded for 15 min. A 2% *w*/*v* solution of chitosan in 3% *v*/*v* aqueous acetic acid was first prepared, under continuous stirring for 3 h at room temperature. An aqueous 2% *w*/*v* solution of gelatin was afterwards obtained, under continuous stirring at 60 °C.

The mass ratio between the inorganic solid and polymer (dry phase) was maintained at 1:1, and the prospective solutions and powders were vigorously stirred until total homogenization. The obtained mixture was cross-linked with aqueous glutaraldehyde 1 wt.%, poured into cylindrical forms, and maintained over night at 4 °C. After reticulation, the sample was soaked in distilled water and maintained for several hours, for the removal of free glutaraldehyde. This process was repeated until a negative result was obtained on the Fehling test, then the sample was subjected to the freeze-drying process (freezing at 55 °C for 12 h, vacuum at 0.001 mbar for 12 h and heating under vacuum for 24 h up to 35 °C) in order to obtain a porous composite material [37]. The final composite product was denoted as HAp(ESM)_CS_Gel_BA. The samples containing only individual parts of the final scaffold were also subjected to the same freeze-drying process. These samples were chosen in order to better understand their influence on porosity, swelling degree and eventual biocompatibility of the resulting material.

### 2.4. Morphological and Structural Characterization

The constituent mineralogical phases from the synthesized HAp(ESM) and their crystallinity degree were analyzed using an X-ray PANalytrical Empyrean diffractometer (Malvern PANalytical, Bruno, Nederland) in Bragg-Brentano geometry, containing an X-ray tube with Cu anode (λCuKα = 1.541874 Å). The X ray diffraction (XRD, Malvern PANalytical, Bruno, Nederland) patterns were registered in the range of 10–80° 2θ angles, with an acquisition step of 0.02° and an acquisition time of 100s in 1D detector mode. The scanning electron microscopy (SEM, Thermo Fisher, Eindhoven, The Netherlands) micrographs were performed with an Inspect F50 microscope coupled with an energy dispersive spectrometer (EDS) (Thermo Fisher—formerly FEI, Eindhoven, The Netherlands), in order to determine the surface and cross-section morphologies and elemental composition. The investigation by Fourier transform infra-red spectroscopy method (FT-IR) of the synthesized scaffold involved the analysis of small quantities of samples with a Nicolet iS50R spectrometer (Thermo Fisher, Waltham, MA, USA). The measurements were performed at room temperature, using the total reflection attenuation module (ATR, Thermo Fisher, Waltham, MA, USA), and 32 scans of the samples between 4000 and 400 cm^−1^ were performed at a resolution of 4 cm^−1^.

### 2.5. Swelling Ratio and Open Porosity Evaluation

To obtain crucial information about the swelling ratio of the scaffolds based on chitosan (CS), chitosan + gelatin (CS_Gel) and of the final inorganic-organic composite HAp(ESM)_CS_Gel_BA, 1 L of SBF (simulated body fluid) solution was first prepared. The ionic species concentration (mmol L^−1^) used correspond to the concentrations of ionic species in blood plasma, according to Kokubo’s recipe, and are described elsewhere [38,39]. The preparation of the SBF solution was performed by following the standard procedure. The first step consists of weighing the ionic species precursors. Afterwards, the weighed precursors are added in a volumetric flask into a water bath at 37 °C so they dissolve completely. The second step consists of verifying the pH of the solution which must be situated between 7.2–7.4. The third step consists of filling the flask with distilled water, continuously checking the temperature. In the last step, the flask is taken out of the water bath, maintained at room temperature for a few minutes and inserted into a refrigerator at 4 °C.

The swelling ratio of the samples was determined by the complete immersion of small cylinders with 5 mm diameter in 5 mL of SBF, maintained at 37 °C for different time periods, followed by their weighing (*W_t_*). Previously, the dry samples were also weighed, in order to determine their initial mass (*W_i_*). With the help of the gravimetric method, the swelling ratio was calculated with Formula (1):

(1) $$\mathrm{Swelling}\;\mathrm{ratio}\;\%=\frac{W_{t}-W_{i}}{W_{i}}\cdot 100$$

where *W_i_* = the initial weight of the sample, *W_t_* = the weight of the inflated sample at time t.

Open porosity was determined using liquid displacement method, in ethanol [40]. Small fragments of scaffold were placed in a cylinder containing a *V*_1_ volume of ethanol. The volume resulting after the scaffold immersion was measured and noted *V*_2_. The scaffold parts were afterwards removed, and the final volume of ethanol from the graduated cylinder was measured and noted V_3_. The porosity degree was calculated using Formula (2):

(2) $$\mathrm{Porosity}\;\%=\frac{V_{1}-V_{3}}{V_{2}-V_{3}}\cdot 100$$

### 2.6. In Vitro Cytotoxicity Assays

#### 2.6.1. Cell Proliferation Assay (MTT)

With the help of this colorimetric quantitative approach, the cellular viability and the cytotoxicity of the obtained scaffolds can be determined. This method is based on the reduction of a yellow tetrazolium salt—MTT (3-(4,5-dimethylthiazol-2-yl)-2,5-diphenyltetrazolium bromide) to its insoluble dark blue formazan. The reduction is carried out by mitochondrial enzymes and it is a sign of cellular/mitochondrial integrity. The formazan can be solubilized with isopropanol, dimethilsulfoxide or other organic solvent. The optical density of solubilized formazan is evaluated spectrophotometrically, and an absorbance–colorant concentration–active cell number function is obtained.

The MTT analysis was performed on GM0047 amniotic fluid stem cell line, purchased from Coriell Institute (Kenton, NJ, USA) and cultivated at the Faculty of Veterinary Medicine, Department of Biochemistry (Bucharest, Romania). These cells were cultivated in Dulbecco’s Modified Eagle’s Medium (DMEM, Sigma-Aldrich, Missouri, MO, USA) supplemented with 10% fetal bovine serum and 1% antibiotics (penicillin and streptomycin) and changed twice a week. A dedicated MTT assay kit (Vybrant^®^ MTT Cell Proliferation Assay Kit, Thermo Fischer Scientific, Massachusetts, MA, USA) was used. The stem cells were cultivated in 96-well plates, with a seeding density of 3000 cells/well in the presence of the synthesized materials (CS, CS_Gel, HAp(ESM)_CS_Gel_BA), for 72 h. The analyzed samples were previously cut in small cylinders with 5 mm diameter with similar mass. Afterwards, 10 μL (12 mM) of MTT were added and the cells were incubated for 4 h at 37 °C. After the incubation, 100 μL SDS-HCl solution (1 mg Sodium Dodecyl Sulphate + 10 mL HCl, 0.01 M) was added dropwise, while the content of the wells was vigorously shaken to help the dissolution of the newly formed formazan crystals. The optical density (OD) of solubilized formazan reading was performed after one hour with the Infinite M200 spectrophotometer (TECAN, Männedorf, Switzerland) at 570 nm [41].

#### 2.6.2. Oxidative Stress Assessment (GSH)

This test was performed on amniotic fluid stem cells (AFSC) cultivated in 96-well plates at a density of 3000 cells/well in 300 μL DMEM enhanced with 10% fetal bovine serum and 1% antibiotics (penicillin and streptomycin). After 24 h, the synthesized materials (CS, CS_Gel and HAp(ESM)_CS_Gel_BA) were added into the wells and incubated for 72 h. The analyzed samples were previously cut in small cylinders with 5 mm diameter with similar mass. The GSH-Glo Glutathione Assay kit (Promega, Fitchburg, WI, USA) was used. This kit determines the quantity of GSH (glutathione) formed by the cells and converted into oxidized glutathione afterwards. The amount of transformed glutathione is directly related to the amount of glutathione S-transferase enzyme (GST) that catalyzes the oxidation, also associated with the creation of a luminescent compound termed luciferin. When the glutathione production is inhibited, the light emission is poor, so oxidative stress is high. When the light emission is more intense, more glutathione has been produced so the cells are less stressed.

The first step of the experimental procedure consisted of the addition of 100 μL 1X GSH-Glo Reagent, followed by incubation at 37 °C for 30 min. In the second step, 100 μL Luciferin Detection Reagent was added and incubated at 37 °C for another 15 min. When the time passed, the medium was homogenized and a luminometer (Microplate Luminometer Centro LB 960, Berthold, Bad Wildbad, Germany) was used for the analysis of the 96-well plate [41].

#### 2.6.3. Evaluation of Cell Morphology

The biocompatibility of the synthesized porous materials (CS, CS_Gel, HAp(ESM)_CS_Gel_BA) was additionally evaluated with the help of fluorescence microscopy, using a cell tracker for long-term tracing of living cells, called RED CMTPX fluorophore (Thermo Fischer Scientific, Massachusetts, MA, USA). The synthesized materials were added in the respective cell cultures along with the CMTPX tracker. After 5 days, the morphology and viability of the AFSCs were evaluated. The CMTPX fluorophore was added in the culture medium at a final concentration of 5 μM. The mediums were incubated for 30 min so the dye penetrated the cells completely. After the incubation period, the cells were washed with PBS (phosphate buffer saline) and analyzed by fluorescent microscopy. The photomicrographs were taken with Olympus CKX 41 digital camera driven by CellSense Entry software (Olympus, Tokyo, Japan) [41].

### 2.7. Statistical Analysis

All the experiments were done in triplicates. Data is represented as mean ± standard deviation (S.D.). The graphs and statistical analysis were performed using MS Excel software. Data was compared using one-way analysis of variance (ANOVA), followed by a two tails *t*-test with Bonferroni post-hoc correction. Values of *p* < 0.05 were considered statistically significant.

## 3. Results and Discussion

### 3.1. Hydroxyapatite Characterization

**Figure 1:**
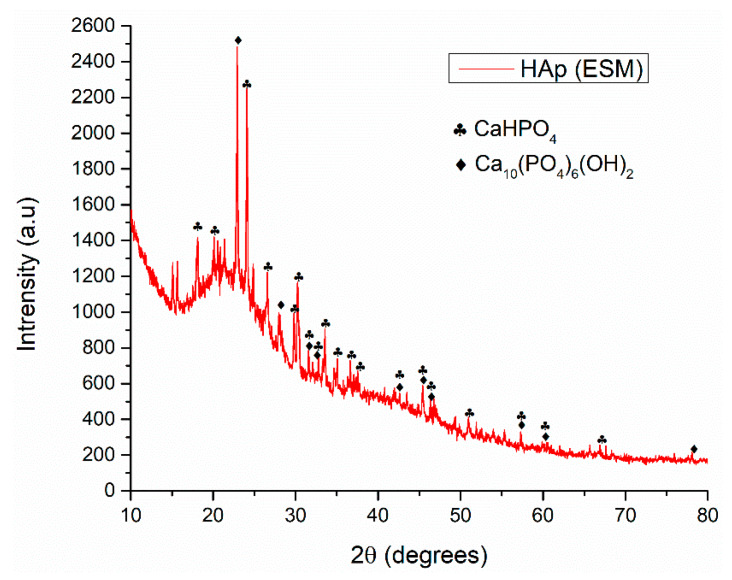
XRD analysis of microwave synthesized hydroxyapatite on ESM.

The X ray diffraction pattern presented in Figure 1, corresponding to the ESM impregnated with the hydroxyapatite precursors and subjected to microwave treatment, highlights the existence of two mineralogical phases that nucleated on the bio-template, respectively hydroxyapatite (HAp, Ca_10_(PO_4_)_6_(OH)_2_) and monetite, known as dicalcium phosphate anhydrous (DCPA, CaHPO_4_). Even though less studied, the latter has found great application recently as an essential component of some bone cements due to its capability to resorb in vivo more rapidly than most of the calcium phosphates, facilitating the implant substitution with the newly formed tissue. Additionally, it is a well-known precursor phase in the synthesis of HAp [42,43].

The identified peaks are sharp and intense, specific to well-crystalized compounds, but the presence of a fairly big halo at small 2θ angles suggests the existence of a certain amount of retained amorphous phases, probably due to the organic ESM support. The majority of the peaks are particular to both hydroxyapatite and monetite phases. According to ASTM (American Society for Testing and Materials), the diffraction maxima identified for hydroxyapatite correspond to the diffraction planes (012), (111), (112), (252), (022), (113), (222) and (133) of hexagonal crystallized hydroxyapatite. In the case of the synthesized monetite, the identified diffraction peaks correspond to the diffraction planes (011), (1−11), (210), (−120) and (−123) of triclinic crystallized monetite.

**Figure 2:**
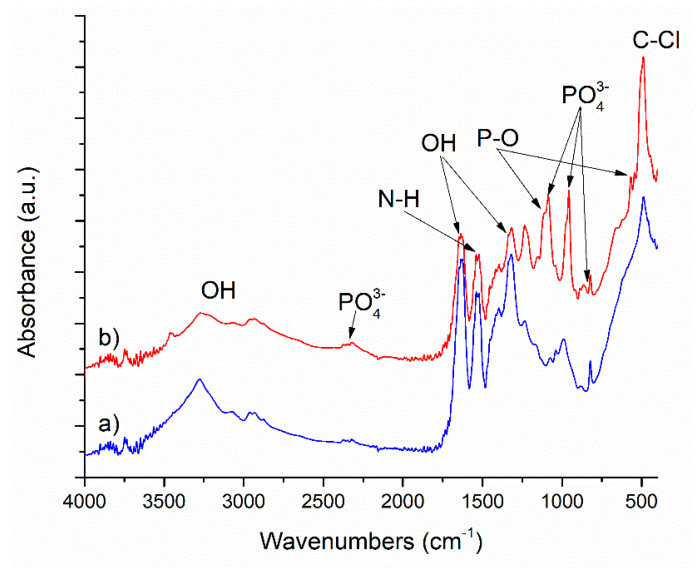
FT-IR spectra for the ESM: (**a**) before and (**b**) after the microwave thermal treatment.

FT-IR spectroscopy was first used to determine the chemical groups present in the raw ESM and the hydroxyapatite prepared using ESM, after microwave treatment. FT-IR spectra for the ESM before and after the thermal treatment are presented in Figure 2. Similar absorbance bands are identified in both spectra, with some particularities especially regarding their absorbance intensities and the appearance of new groups after thermal treatment. Hence, after impregnation with hydroxyapatite precursors and exposure of ESM to microwaves, calcium phosphates were formed on the surface of ESM fibers (in correlation with the XRD results), including hydroxyapatite. This is observed in FT-IR by a decrease in absorbance of the N–H amidic band from 1522 cm^−1^, specific to the ESM fibers proteins, and secondly by the apparition of characteristic asymmetric stretching vibrations of phosphate functions between 950–1050 cm^−1^. The bands from around 567 and 957 cm^−1^ correspond to the V1 stretching mode of the P–O bond, also indicate the hydroxyapatite formation. Both materials present high absorption bands between 3000–3500 cm^−1^, characteristic to the amino functional groups and hydroxyl (O–H) stretching mode. The band around 1318 cm^−1^ also corresponds to O–H stretching. The bands around 1634 and 1539 cm^−1^ correspond to amidic group vibrations and to N–H bonds. The band around 488 cm^−1^ corresponds to C-halogen stretching, most probably C–Cl (from the detachment procedure of ESM from the eggshell) [29].

**Figure 3:**
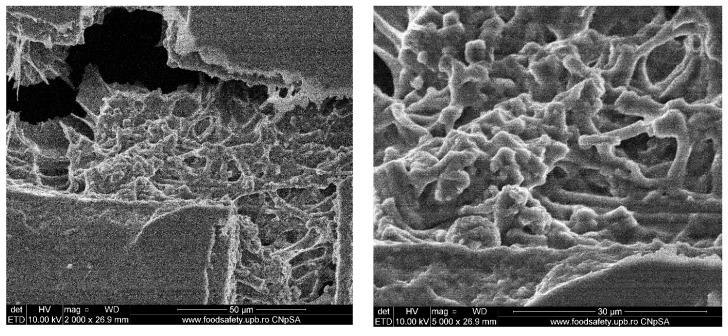
SEM micrographs of impregnated ESM after microwave thermal treatment (general aspect on the **left** and an up-close detail on the **right**).

In Figure 3, SEM micrographs of ESM after microwave thermal treatment and before grinding are presented. The first micrograph confers an overall image of the microwave treated ESM in which there is a crack that unveils the interwoven structure. In the next micrograph, the interwoven structure of the ESM can be seen from up close, completely covered by the hydroxyapatite formation.

The nucleated hydroxyapatite does not have a uniform dimensional distribution; agglomerates with spherical morphology and a diameter of a few microns, as well as bigger structures with dimensions up to 15 μm can be observed. The obtained results differ from the ones reported in the study by Sabu et al., which presents HAp nanocrystallites [29]. This can probably be attributed to an imbalance between the nucleation and crystal growth rates, in this case the crystal growth rate being too high in comparison to the nucleation rate. It is known that a high nucleation rate, generating small crystals in the final product, can be obtained by fast heating processes, such as microwave heating, but it was found that higher heating rates influence not only the rate of nucleation but also the rate of crystal growth [44]. Even though the heating parameters and the ratio between nucleation and crystal growth can be still improved, the SEM micrographs show that the ESM structure is maintained during hydroxyapatite synthesis, conferring an excellent support for cellular adhesion.

### 3.2. Composite Scaffold and Saffold Component Characterization

**Figure 4:**
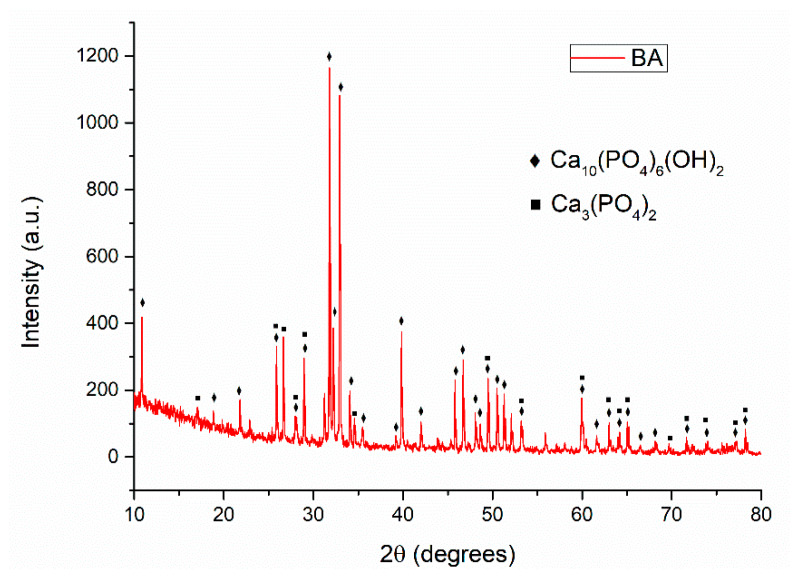
XRD analysis of calcined bovine bone ash (BA).

The X-ray diffraction pattern presented in Figure 4, corresponding to the calcined bovine bone ash (BA) at 750 °C for 6 h highlights the existence of two mineralogical phases, respectively hydroxyapatite and tricalcium phosphate (TCP), a calcium salt of phosphoric acid with the chemical formula Ca_3_(PO_4_)_2_, exhibiting great bioactivity, as well as high degradability [45]. Along with HAp (ESM), the addition of BA containing TCP to the final composite scaffold will confer increased biocompatibility, since recent studies have revealed the ability of HAP and β-TCP mixtures to stimulate the osseogenic differentiation of mesenchymal stem cells, to increase cell adhesion, and enhance mechanical properties [46,47].

**Figure 5:**
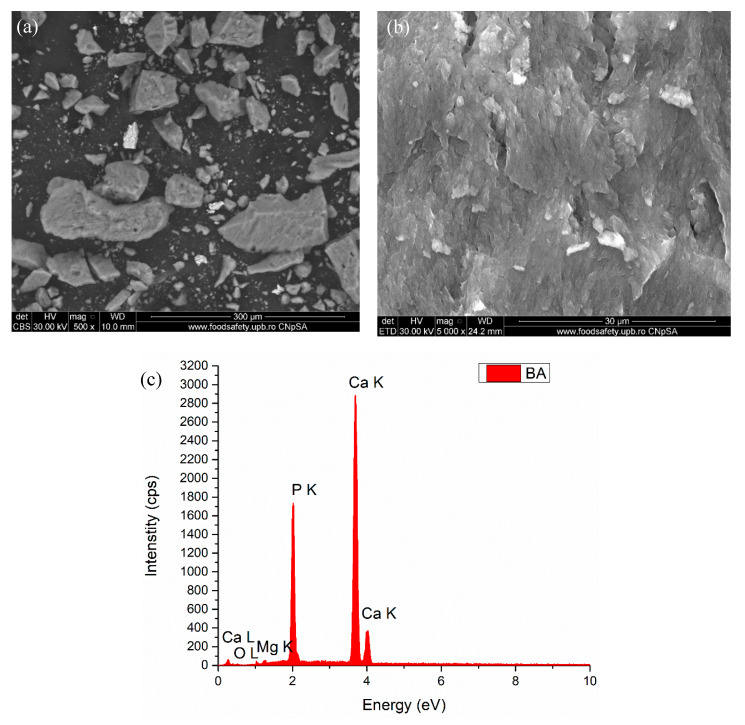
SEM micrographs (**a,b**) and EDS analysis spectrum (**c**) of bovine bone ash (BA).

In Figure 5 are presented two SEM micrographs of the calcined bone (bone ash) and the EDS associated spectrum. In the first micrograph, a wide range of particle sizes and shapes characterize the bone ash powder; the particles have asymmetrical shapes with edges and corners, with dimensions ranging from a few microns to 100–150 μm. These asymmetrical morphologies can be a result of the intrinsic grinding of the burnt bone during thermal treatment and are related with the type, age and nutrition of that exact animal from which the bone was obtained, as previously reported by Bahrololoom et al. [48]. The resulting ash was free of any amorphous organic materials, which were most probably burnt at about 400 °C and completely eliminated at 750 °C, resulting in a white powder. The EDS spectrum of the bone ash presents all the characteristic elements for calcium phosphates along with magnesium, which is commonly present in the elemental composition of bone [49].

**Figure 6:**
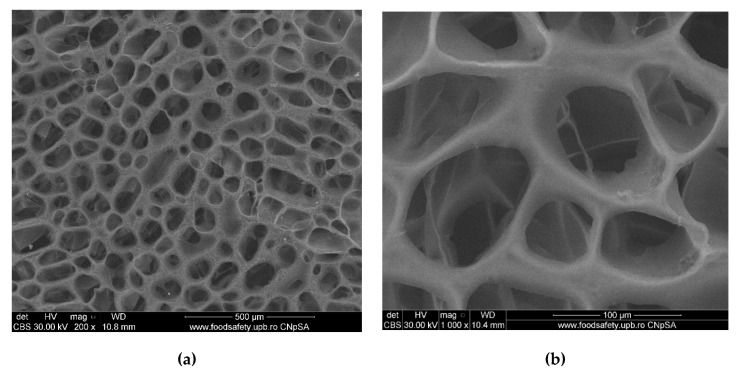
SEM micrographs (**a**-general aspect, **b**-an up-close detail) of freeze-dried chitosan sample (CS).

**Figure 7:**
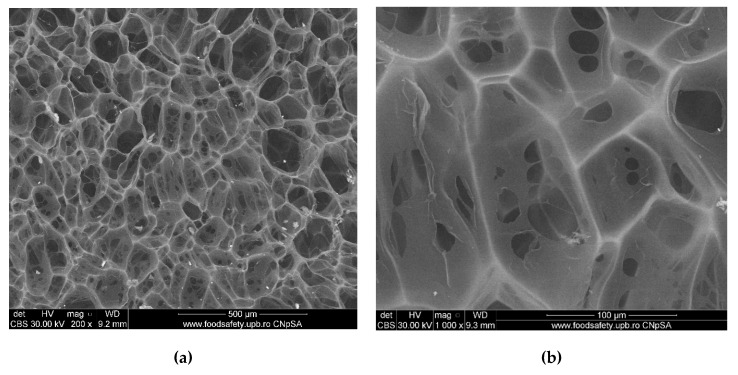
SEM micrographs (**a**-general aspect, **b**-an up-close detail) of freeze-dried chitosan and gelatin sample (CS_Gel).

Figure 6 presents the SEM micrographs of the freeze-dried chitosan sample. Both micrographs show its highly porous nature, characteristic to this drying method, with rounded, interconnected pores of 30–60 μm in diameter. With the addition of gelatin to chitosan, the microporous structure is maintained, as presented in Figure 7, but some small fibers are observed to grow on the chitosan surface and in some of the pores, conferring compactness. The porous structures of chitosan and gelatin play an important role in bone formation, because they allow vascularization, as well as migration and proliferation of osteoblasts and mesenchymal cells, and therefore, they can be successfully used as a support in the final scaffold for tissue engineering [50,51].

**Figure 8:**
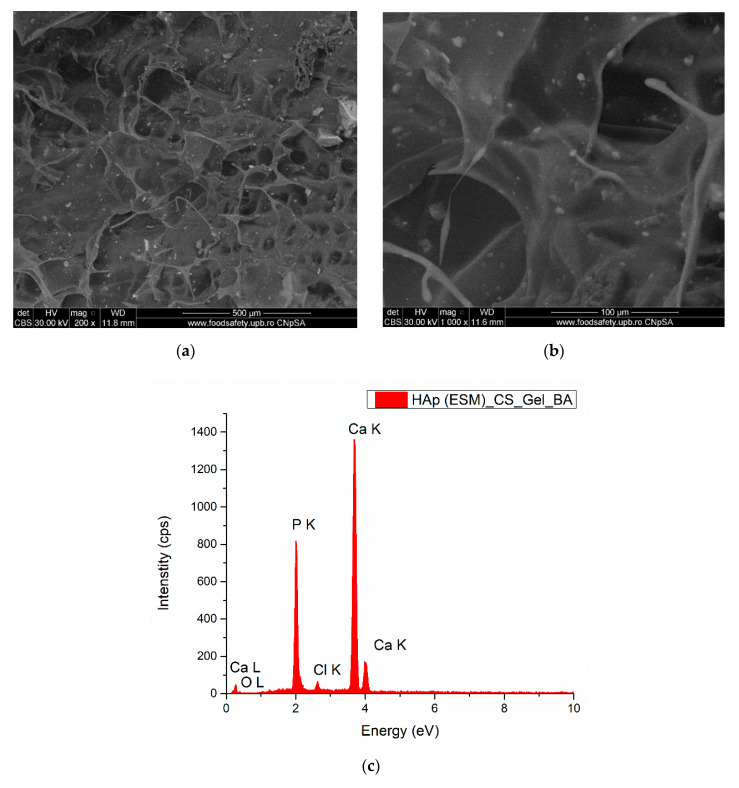
SEM micrographs (**a**,**b**) and EDS spectrum (**c**) of the composite scaffold—Hap(ESM)_CS_Gel_BA.

The SEM micrographs and EDS spectrum of the final HAp(ESM)_CS_Gel_BA biocomposite scaffold are presented in Figure 8. Both micrographs show a more compact structure of the scaffold, with fewer, larger pores, compared with the previous CS and CS_Gel samples, due to the addition of the inorganic components. The uniform distribution of hydroxyapatite and bone ash powders can be observed. The pores are bigger than 100 μm, with these dimensions being excellent for bone regeneration, since osteoblasts have a preference for larger pores (100–200 μm) for regenerating mineralized bone after implantation. These dimensions allow the infiltration of macrophages and other cells involved in colonization, along with bacteria elimination and in vivo vascularization. In the case of pore size below < 100 μm, usually fibrous tissue or non-mineralized osteoid are formed [5]. The EDS spectrum confirms the presence of calcium phosphates elemental components.

**Figure 9:**
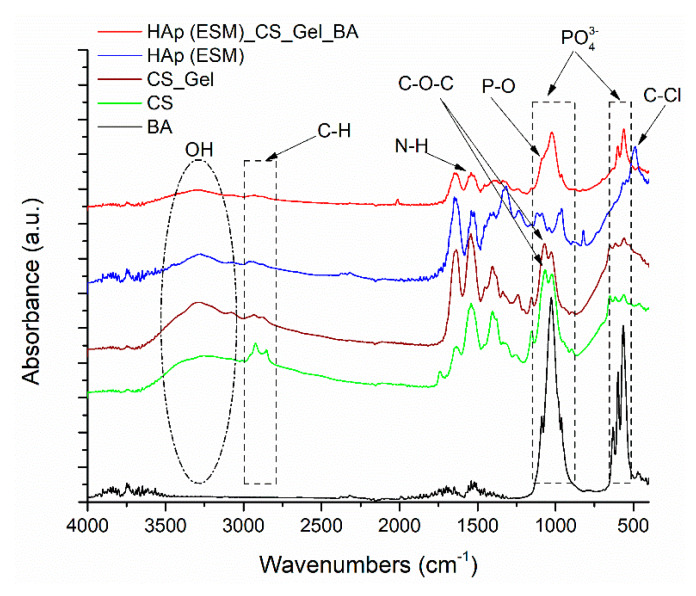
FT-IR spectra for the composite scaffold - HAp(ESM)_CS_Gel_BA.

The FT-IR spectra for the individual components and the final composite scaffold Hap(ESM)_CS_Gel_BA are presented in Figure 9. The main goal of this investigation is represented by the identification of all chemical constituents in the final material, along with establishing the possible interactions between their characteristic functional groups.

Hence, the specific bands for CS and Gel, namely the stretching bands of –NH_2_ and –OH functional groups around 3256 cm^−1^, the band from 2922 cm ^−1^ corresponding to C–H stretching mode and the bands from 1635, 1541 and 1403 cm^−1^, which indicate amide III (C–N), amide II (N–H) and amide I (C=O) groups, suffer a decrease in absorbance in the biocomposite, mostly because of the addition of the inorganic phase, diminishing part of the signal [52,53]. The calcium phosphates (including hydroxyapatite) are also identified in the final scaffold and represented by their characteristic bands around 959 and 821 cm^−1^, corresponding to the P–O bond [36].

**Figure 10:**
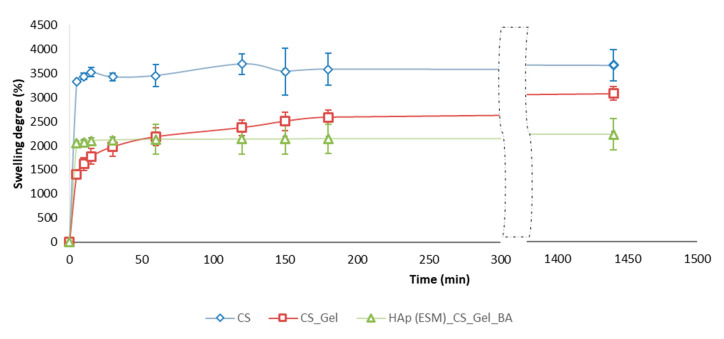
SBF swelling degree evaluation (presented as mean ± S.D. of three replicates).

**Figure 11:**
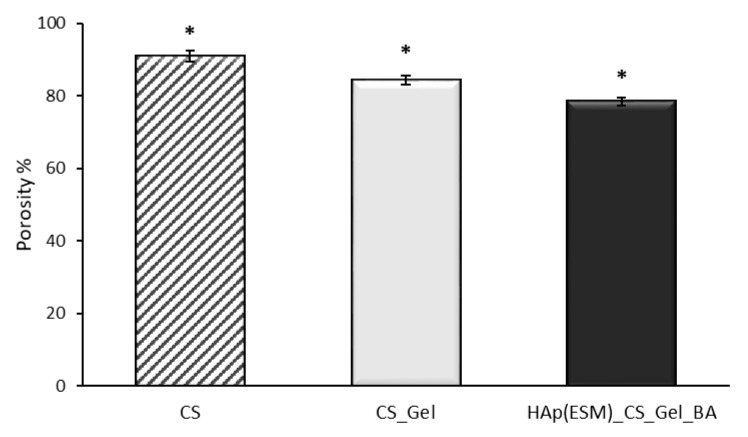
Open porosity % evaluation (presented as mean ± S.D. of 3 replicates); * means a value *p* < 0.005, obtained by single factor ANOVA test, followed by a post-hoc Bonferroni corrected two tails *t*-test (n = 3).

In Figure 10, the dynamics of SBF absorption in time for the freeze-dried CS, CS_Gel and Hap(ESM)_CS_Gel_BA porous samples are presented. The swelling degree of a scaffold is strongly related to the open porosity (Figure 11) and is an important parameter, because a high absorption rate allows all the necessary ions to be acquired from the medium, in order to faster regenerate the damaged bone.

A high fluid uptake ability can be easily observed in the cases of CS and CS_Gel polymeric samples, which is due to their great hydrophilicity and swelling properties. Hence, the CS freeze-dried porous scaffold presented the best results (~3500%), followed closely by CS_Gel (due to the dissolving of gelatin the pore size decreased, leading to less SBF absorption), and finally HAp(ESM)_CS_Gel_BA. This may be attributed to a potential reaction between calcium and phosphate and the hydrophilic –COOH or -NH_2_ groups, decreasing the hydrophilicity of gelatin [54]. The swelling degree results are in accordance wih the determined open porosity and SEM micrographs. There was a statistically significant difference between the analyzed samples, determined by single-factor ANOVA (F(2,6) = 72.8139, *p* = 0.00006), followed by a post-hoc Bonferroni corrected two tails *t*-test (n = 3).

**Figure 12:**
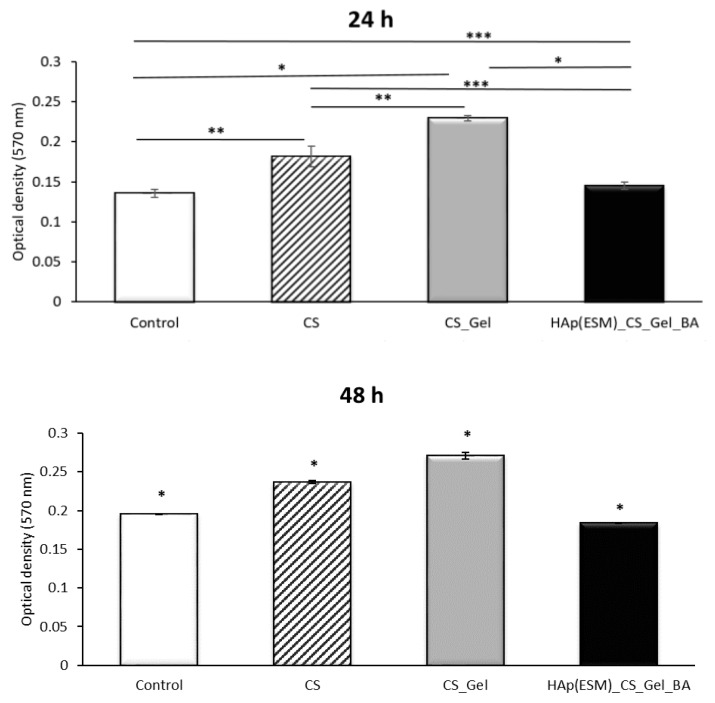

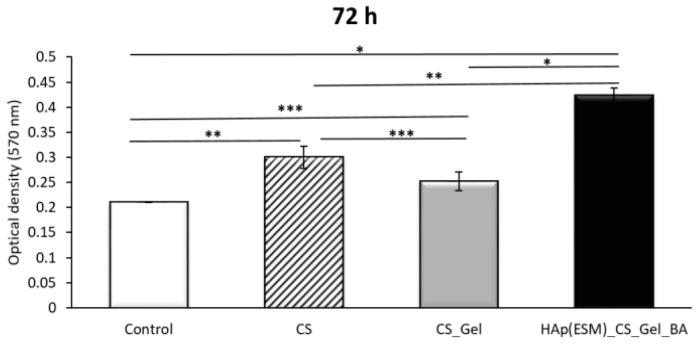
MTT analysis results after 24, 48 and 72 h for the CS, CS_Gel and HAp(ESM)_CS_Gel_BA porous samples (presented as mean ± S.D. of 3 replicates); * means a value *p* < 0.0005, ** means a value *p* < 0.005, *** means a value *p* < 0.05.

The results of the MTT assay after 24, 48 and 72 h are presented in Figure 12. The information presented in the graph shows the fact that all synthesized materials exhibited good biocompatibility and non-cytotoxicity with respect to the AFSCs, the absorbance values for all porous scaffolds being statistically significantly higher than the control (AFSCs without added materials). Moreover, the absorbance values increased over time; therefore, the synthesized materials were also able to stimulate cellular proliferation, the best results in optical density at 570 nm being registered after 72 h for the HAp(ESM)_CS_Gel_BA final biocomposite scaffold. This is probably a result of the bioactive nature of the added calcium phosphates, coupled with the high surface area determined by the porous morphology of the scaffolds.

**Figure 13:**
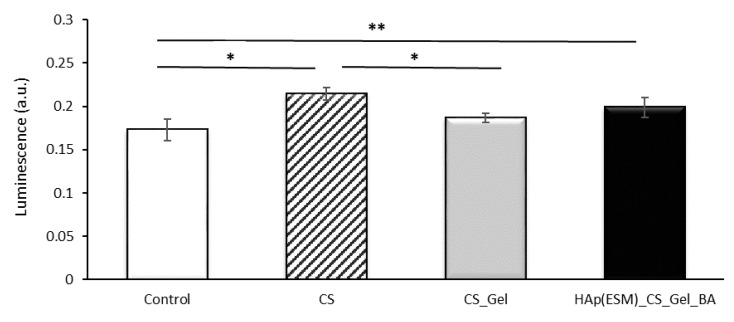
GSH analysis results after 24 h for the CS, CS_Gel and HAp(ESM)_CS_Gel_BA porous samples (presented as mean ± S.D. of 3 replicates); * means a value *p* < 0.005 Control versus CS and CS versus CS_Gel; ** means a value *p* < 0.05 Control versus HAp(ESM)_CS_Gel_BA.

The oxidative stress produced by the materials on the AFSCs was tested via the GSH method after 24 h, and the luminescence results are presented in Figure 13. When compared with the control luminescence level (obtained after the evaluation of cells development without the addition of other materials), a statistically significant higher glutathione level is registered in the cases of CS and HAp(ESM)_CS_Gel_BA. Overall, no statistically significant lower values were obtained after the interaction of the obtained materials with the cells. The fact that the glutathione synthesis was not inhibited is directly associated with a lower stress level of AFSCs in the presence of the final biocomposite scaffold and its individual components, proving once more their high biocompatibility and non-cytotoxic behavior. The results found statistically significant, determined by single-factor ANOVA (F(3,8) = 10.389, *p* = 0.0039), followed by a post-hoc Bonferroni corrected two tails *t*-test (n = 6), are presented in Figure 13 caption.

**Figure 14:**
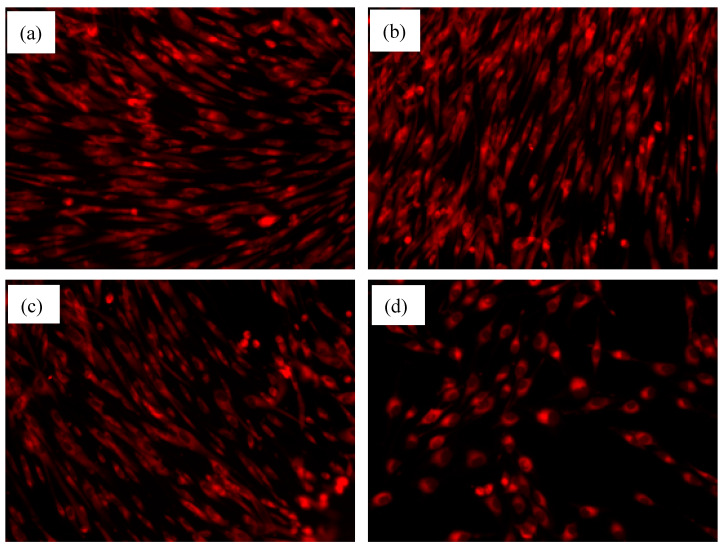
Fluorescence microscopy images of the AFSC interaction with: (**a**) CS, (**b**) CS_Gel, (**c**) HAp(ESM)_CS_Gel_BA, and (**d**) control sample.

The interaction of the synthesized materials with AFSCs was also analyzed by means of fluorescence microscopy, in order to visualize what effect the materials have on cellular morphology through the examination of tubulin filaments. In Figure 14a–c one can observe numerous AFSCs, increased filopodia extension and elongated morphology as compared to the control (Figure 14d, in which the cells present their characteristic spherical morphology, only a few presenting prolongations). This could be associated with an active metabolism and good adhesion of the cells to the substrate. The modification of cellular morphology depends on many factors like the adherence degree to the substrate, cellular stress, osmolality modifications, exposure period and cellular type (in case of cellular differentiation). Due to the elongated cellular morphology characteristic to bone cells, acquired after contact with the material, it is probable that the AFSCs may have been differentiated with respect to cells of osteogenic lineage, a fact previously reported in the literature, but further tests on osteoblast differentiation and mineralization (e.g., Alkaline phosphatase and Alizarin red S) are needed [55].

## 4. Conclusions

The experimental activities reported in this paper propose the synthesis of a new biocompatible composite scaffold based on natural compounds, with low production costs. The hydroxyapatite synthesis using ESM as bio-template generates hexagonally crystallized hydroxyapatite, along with triclinic monetite, while the addition of polymers and the freeze-drying technique used for the scaffold synthesis generates a porous morphology, with uniform distribution of the inorganic powder. The in vitro biocompatibility, porosity and swelling degree evaluation confirm the excellent biocompatibility of the scaffold, the final material proving to be non-cytotoxic, and allowing cellular proliferation, stem cells adhesion and multiplication.
